# A method for isolating and cryopreserving intact mitochondria with improved integrity and functionality

**DOI:** 10.2142/biophysico.bppb-v22.0012

**Published:** 2025-05-16

**Authors:** Arima Okutani, Jannatul Naima, Asaka Ogihara, Taihei Motoichi, Ikuroh Ohsawa, Yoshihiro Ohta

**Affiliations:** 1 Division of Biotechnology and Life Sciences, Tokyo University of Agriculture and Technology, Koganei, Tokyo 184-8588, Japan; 2 Department of Pharmacy, University of Chittagong, Chittagong 4331, Bangladesh; 3 Biological Process of Aging, Tokyo Metropolitan Institute of Gerontology, Tokyo, 173-0015, Japan

**Keywords:** mitochondria, isolation, cryopreservation

## Abstract

Mitochondria isolated from cells are essential tools in biological research. However, many mitochondria are often damaged during the isolation process. Although cryopreservation can greatly improve the usability of isolated mitochondria, it typically leads to significant loss of activity following freezing and thawing. In this study, we present our own techniques for mitochondrial isolation and cryopreservation to overcome these challenges. Our isolation method begins by selectively weakening the plasma membrane through the incorporation of digitonin, under conditions that do not increase membrane permeability. The plasma membrane is then selectively ruptured to release mitochondria. Notably, mitochondria contract within the cell before the plasma membrane ruptures, a process that facilitates their extraction. The isolated mitochondria showed polarized inner membranes in approximately 90% of the population. Compared to mitochondria isolated by homogenization, they retained more intermembrane space proteins and exhibited greater outer membrane integrity. For cryopreservation, rapid thawing was critical to maintaining mitochondrial activity after freeze-thaw cycles. When thawing was completed in under 1.5 minutes, the proportion of polarized mitochondria decreased by only about 10%. These findings suggest that our isolation and cryopreservation protocols are promising for applications requiring intact, functional mitochondria.

## Significance

Isolated mitochondria are essential tools in mitochondrial research. However, conventional isolation procedures often cause structural damage, making it difficult to obtain intact mitochondria. Furthermore, freezing and thawing typically result in significant activity loss, limiting their utility in experimental studies. This paper presents a new mitochondrial isolation method that substantially reduces damage to both the inner and outer membranes. In addition, we introduce a cryopreservation technique that enables stable freeze-thaw cycles with minimal loss of mitochondrial activity. We expect that mitochondria prepared using these methods will facilitate further advances in mitochondrial research.

## Introduction

Mitochondria are essential organelles that play key roles in numerous cellular processes, including energy metabolism, signal transduction, cell death, and the production of reactive oxygen species (ROS). Dysfunctional mitochondria have been implicated in a wide range of diseases, making them a major focus of biomedical research. Isolated mitochondria provide an ideal model for studying mitochondrial function, as their experimental environment can be precisely controlled. Important discoveries made using isolated mitochondria include the development of the chemiosmotic theory [1], investigations into mitochondrial permeability transition [2], observations of matrix density changes in response to metabolic activity [3], analyses of inner membrane remodeling during apoptosis [4], and studies on ROS generation [5,6].

Numerous recent studies have reported that administering isolated mitochondria can modulate cellular functions. For example, isolated mitochondria have been shown to attenuate ischemia-reperfusion (I/R) injury in the heart [7] and brain [8], improve recovery from spinal cord injury [9], reduce hypoxic pulmonary hypertension [10], suppress cancer cell proliferation [11], ameliorate cognitive impairment [12], and modulate immune cell responses [13]. Although the precise mechanisms by which isolated mitochondria influence cellular functions remain unclear, McCully et al. [7] demonstrated that intact, highly functional mitochondria effectively reduce cardiac I/R injury. In contrast, mitochondrial fragments and freeze-thawed mitochondria were found to be ineffective. These findings highlight the strong need for intact, functional mitochondria in both research and therapeutic applications.

Isolating mitochondria from cells requires disruption of the plasma membrane. Traditional methods typically involve mechanical disruption by homogenization [14] or membrane solubilization using surfactants [15]. However, homogenization can damage both the plasma membrane and mitochondria [16], while surfactants pose a similar risk by gaining access to mitochondria immediately after disrupting the plasma membrane [17]. To preserve mitochondrial integrity, both mechanical disruption and surfactant-based solubilization should be avoided. Furthermore, the ability to freeze and thaw high-quality, functional mitochondria without significant activity loss would enable their distribution to other laboratories, potentially accelerating the broader application of isolated mitochondria in research and therapy.

This study introduces a novel technique, iMIT (intact mitochondria isolation technique), for isolating structurally intact mitochondria. The iMIT method enables the isolation of a mitochondrial population in which the outer membrane remains largely preserved. This is accomplished by first reducing the mechanical strength of the plasma membrane using digitonin—without increasing its permeability—and then selectively disrupting the membrane with gentle mechanical stimulation. In addition, we have optimized the freeze-thaw process to preserve high mitochondrial activity after thawing. The methods developed in this study are expected to significantly advance research into mitochondrial function and support the development of mitochondria-based therapeutic applications.

## Materials and methods

### Materials

ICR female mice (9 weeks old) were purchased from CLEA Japan Inc. (Tokyo, Japan) for mitochondrial isolation. C6 (RCB2854) and HeLa (RCB0007) cells were obtained from the RIKEN BioResource Research Center (RIKEN BRC; Saitama, Japan), and human umbilical vein endothelial cells (HUVECs, JCRB1458) were purchased from the JCRB Cell Bank (Osaka, Japan). Minimum Essential Medium (MEM), Roswell Park Memorial Institute Medium 1640 (RPMI 1640), and MCDB 131 were obtained from Thermo Fisher Scientific (Waltham, MA, USA). Tetramethylrhodamine ethyl ester (TMRE), MitoTracker Red CMXRos, the Mitochondria Isolation Kit for Cultured Cells, and Alexa Fluor Plus 488-conjugated anti-rabbit IgG were also purchased from Thermo Fisher Scientific. Calcein-AM was obtained from Dojindo Laboratories (Kumamoto, Japan), and the CellTiter-Glo Luminescent Cell Viability Assay was purchased from Promega (Madison, WI, USA). The BCA Protein Assay Kit and digitonin were also obtained from Promega. Primary antibodies including anti-Tom20, anti-cytochrome c (Cyt c), anti-adenylate kinase 2 (AK2), anti-citrate synthase (CS), anti-VDAC, anti-COX IV, and anti-β-actin were purchased from Proteintech Group Inc. (Rosemont, IL, USA). Horseradish peroxidase (HRP)-conjugated anti-rabbit IgG was obtained from Santa Cruz Biotechnology, Inc. (Santa Cruz, CA, USA), and ECL Advance Western Blotting Detection System was purchased from GE Healthcare (Chicago, IL, USA). All other chemicals used were of the highest commercially available purity.

### Cell culture

C6 was maintained in RPMI 1640 supplemented with 10% fetal bovine serum (FBS) and 2.0 g/L NaHCO_3_. HeLa was maintained in MEM supplemented with 10% FBS and 2.2 g/L NaHCO_3_. Huvec was maintained in MCDB131 medium supplemented with 10% FBS, 0.03 g/L endothelial cell growth supplement, 5 μg/mL heparin, and 10 mM L-glutamine. All cells were cultured on culture dishes at 37°C in an incubator humidified with a 5% CO_2_ atmosphere.

### Mitochondrial isolation from cultured cells

#### Preparation:

Cells were cultured in 150-mm diameter dishes until approximately 80% confluence was reached. They were washed twice with 10 mL of Tris-isolation buffer (10 mM Tris-HCl, 250 mM sucrose, and 0.5 mM EGTA, pH 7.4).

#### Mitochondrial Isolation Techniques:

Four methods were used for mitochondrial isolation: iMIT, HBM, H-kit, and R-kit.

#### iMIT Procedure:

1. Cells in dishes were incubated with 9 mL of Tris-isolation buffer containing 30 μM digitonin at 4°C for 3 minutes.

2. Following incubation, cells were washed twice with Tris-isolation buffer and incubated in the same buffer at 4°C for 10 more minutes.

3. The cells were detached by gentle pipetting, and the resulting cell suspension was further agitated several times by pipetting.

4. The suspension was centrifuged at 500×g for 10 minutes at 4°C.

5. The supernatant was centrifuged at 3,000×g for 10 minutes at 4°C.

6. The pellet was collected as mitochondrial fraction and resuspended in a Tris-isolation buffer.

#### HBM Procedure:

1. Cells were detached using a cell scraper in a Tris-isolation buffer.

2. The cell suspension was centrifuged at 200×g for 5 minutes at 25°C to remove mitochondria released from broken cells.

3. The pellet was resuspended in 1 mL of ice-cold Tris-isolation buffer and homogenized with a Teflon homogenizer (40 strokes at 4°C).

4. Steps 4 to 6 in the iMIT procedures were used for further centrifugation and mitochondrial pellet collection.

#### H-kit and R-kit Procedures:

Mitochondria were isolated using the Thermo Fisher Mitochondrial Isolation Kit for Cultured Cells using either the homogenization-based (H-kit) or reagent-based (R-kit) method according to the manufacturer’s instructions.

### Mitochondrial isolation from skeletal muscles and liver

#### Preparation:

Mice were euthanized by intraperitoneal injection of 150 mg/kg pentobarbital sodium salt. After confirmation of death, quadriceps and liver tissues were excised, rinsed with phosphate-buffered saline (PBS) (137 mM NaCl, 2.7 mM KCl, 1.5 mM KH_2_PO_4_, 8 mM NaHPO_4_), and stored in PBS at 4°C. The excised tissues were minced into small pieces on ice. The small tissue samples were treated with 5% trypsin at 50 mg/mL in PBS and incubated at 4°C for 30 minutes for skeletal muscle and 15 minutes for liver tissue. The trypsinized suspension was centrifuged at 200×g for 10 minutes at 4°C to remove trypsin and mitochondria released from broken cells, and the pellet was collected.

#### iMIT Procedure:

1. The obtained pellet was resuspended in a Tris-isolation buffer with 15 μM digitonin.

2. This suspension was centrifuged at 200×g for 10 minutes at 4°C to remove excess digitonin.

3. The pellet was then incubated in Tris-isolation buffer at 4°C for 10 minutes and resuspended through gentle pipetting.

4. This suspension underwent centrifugation at 500×g for 10 minutes at 4°C.

5. The supernatant was centrifuged at 3,000×g for 10 minutes at 4°C.

6. The final mitochondrial fraction was obtained by resuspending the pellet in a Tris-isolation buffer.

#### HBM Procedure:

The trypsinized pellet was resuspended and homogenized as described for the HBM of mitochondrial isolation from cultured cells. The mitochondrial fraction was obtained as a pellet following centrifugation at 3,000×g for 10 minutes at 4°C.

### Freeze-thaw treatment of isolated mitochondria

Prior to freezing, 0.1 or 1.0 mL of mitochondrial suspension (approximately 500 μg protein/mL) was dispensed into 3.0 mL tubes. The tubes were then frozen in liquid nitrogen and stored at either –196°C or –80°C, depending on the experimental requirement. Frozen mitochondria were thawed either rapidly under running water at 20°C or slowly on ice.

### Fluorescence staining

For fluorescence staining with calcein and TMRE, C6 cells cultured on glass-bottom dishes (GBDs) were incubated with 500 nM calcein-AM and 20 nM TMRE for 10 minutes at 37°C in HEPES-buffered saline (10 mM HEPES, 120 mM NaCl, 4 mM KCl, 0.5 mM MgSO_4_, 1 mM NaH_2_PO_4_, 4 mM NaHCO_3_, 25 mM glucose, 1.2 mM CaCl_2_, and 0.1% BSA, pH 7.4). After staining, cells were washed with ice-cold Tris isolation buffer containing 20 nM TMRE and maintained in the buffer until detachment from the dish. For TMRE staining of isolated mitochondria, mitochondria were adsorbed onto GBDs by centrifugation at 100×g for 5 minutes at 4°C [18], and then incubated with 10 nM TMRE in Tris isolation buffer containing 0.33 mg/mL BSA for 10 minutes at 25°C.

To evaluate the integrity of the outer and inner mitochondrial membranes, HeLa cells cultured on dishes were stained with 500 nM MitoTracker Red in MEM for 30 minutes at 37°C, followed by mitochondrial isolation. The isolated mitochondria were adsorbed onto GBDs and incubated with anti-Tom20 primary antibody (1:50 dilution) for 1 hour at 25°C. After washing with Tris isolation buffer, mitochondria were incubated with Alexa Fluor Plus 488-conjugated secondary antibody (1:250 dilution) for 1 hour at 25°C. Before observation, mitochondria were washed three times with Tris isolation buffer.

### Fluorescence imaging

For calcein fluorescence, excitation was performed using a 20-nm bandpass filter centered at 480 nm, and emission was collected using a bandpass filter spanning 515–550 nm. For TMRE, excitation was achieved with a 15-nm bandpass filter centered at 535 nm, and emission was collected using a long-pass filter above 580 nm.

To detect MitoTracker Red and Alexa Fluor Plus 488 signals in mitochondria, we used the multicolor gSTED module of the Leica TCS SP8 STED 3× microscope (Leica Microsystems, Wetzlar, Germany). The system was equipped with an HC PL Apo CS2 100×/1.40 OIL objective, a white light laser as the pulsed excitation source, a 660 nm depletion laser, and hybrid detectors with a time gate of 0.5–6.5 ns. Alexa Fluor Plus 488 was excited at 488 nm, and fluorescence was collected between 500 and 550 nm. MitoTracker Red was excited at 580 nm, and fluorescence was collected between 590 and 650 nm.

### Protein assay

The protein content was determined using a BCA assay kit with bovine serum albumin as the standard.

### ATP assay

Isolated mitochondria were suspended at a concentration of 0.1 mg protein/mL in Tris-KCl buffer (10 mM Tris, 110 mM sucrose, 70 mM KCl, and 0.5 mM EGTA, pH 7.4) supplemented with 10 mM malate, 5 mM glutamate, and 10 mM KH_2_PO_4_. In some experiments, 5 μM oligomycin, an inhibitor of FoF_1_-ATPase, was added to the suspension. ATP synthesis was initiated by adding 0.1 mM ADP, followed by incubation at 25°C for 10 minutes. The mitochondrial suspension was then centrifuged at 8,000×g for 10 minutes at 4°C, and the supernatant was collected. ATP concentration in the supernatant was measured using a commercial luminescent assay kit (CellTiter-Glo Luminescent Cell Viability Kit; Promega, Madison, WI, USA). Bioluminescence was detected with a microplate reader (SpectraMax iD3; Molecular Devices, San Jose, CA, USA).

### Western blotting analysis

Mitochondria isolated from C6 cells were suspended in sodium dodecyl sulfate polyacrylamide gel electrophoresis (SDS-PAGE) sample buffer (0.1 M Tris-HCl, 20% glycerol, 1 mM dithiothreitol, 4% SDS, and 0.004% bromophenol blue, pH 6.8) at a concentration of 4 mg protein/mL. The samples were denatured by heating at 98°C for 10 minutes and then divided into 2 to 6 portions to compare protein levels in mitochondrial subcompartments between mitochondria isolated by homogenization (Hmit) and those isolated by iMIT (Imit). One portion was used to detect citrate synthase (CS) as a loading control, and the remaining portions were used to detect target proteins. Equal amounts of protein (40 μg) were loaded into each lane of a 12% SDS-polyacrylamide gel, separated by electrophoresis, and transferred onto nitrocellulose membranes.

Each lane on nitrocellulose membranes was subjected to both Ponceau S staining and western blotting. For Ponceau S staining, membranes were incubated in 0.1% (w/v) Ponceau S in 5% acetic acid for 30 minutes at 25°C with gentle agitation. After washing with pure water (Milli-Q, Merck Millipore, Burlington, MA, USA), stained bands were visualized using a CCD camera.

For western blotting, membranes were incubated with primary antibodies—anti-VDAC, anti-AK2, anti-COX IV, anti-cytochrome c, anti-β-actin, or anti-CS—diluted at 1:5000 in PBS containing 0.1% (w/v) Tween 20, for 1 hour at 25°C with gentle agitation. After washing with PBS-Tween, membranes were incubated with horseradish peroxidase (HRP)-conjugated anti-rabbit IgG (1:10,000 dilution) for 1 hour at 25°C. Target proteins were detected using ECL Advance reagent and imaged with either a Typhoon 8600 molecular dynamics system (BioSurplus; San Diego, CA, USA) or a chemiluminescence imaging system (Luminograph II; ATTO, Tokyo, Japan).

For each lane, the integrated luminescence intensity of the target protein band was measured [19], and the total Ponceau S staining intensity was used to estimate the total protein loaded. Image analysis was performed using MetaMorph software (Version 7.8; Universal Imaging, Downingtown, PA, USA). The intensity of each target protein band was normalized to the total Ponceau S staining intensity and denoted as L_target protein_. To evaluate the relative abundance of each target protein, the ratio of L_target protein_ to L_CS_ was calculated using mitochondria from the same preparation, where L_CS_ represents L_target protein_ when the target protein is citrate synthase (CS). To compare protein levels between mitochondria isolated with iMIT and mitochondria isolated with HBM, the ratio of L_target protein_/L_CS_ in Imit to that in Hmit was calculated and referred to as R_target protein_.

### Statistical analysis

The data in the present study were obtained from at least three independent samples and were expressed as the mean±standard error of the mean (SEM). Data were analyzed by a two-tailed analysis of variance (ANOVA), followed by the Student-Newman-Keuls test. The difference was considered statistically significant at p<0.05.

## Results

### Assessment of plasma membrane permeability, mitochondrial function, and morphology during the iMIT procedure

To isolate intact mitochondria, we aimed to weaken the plasma membrane using a low concentration of detergent and disrupt it through gentle pipetting, thereby minimizing mitochondrial exposure to the detergent. To verify this approach, C6 cells were double-stained with calcein and TMRE. Calcein fluorescence was used to assess plasma membrane permeability, while TMRE fluorescence was used to evaluate mitochondrial membrane potential and morphology.

**Figure 1:**
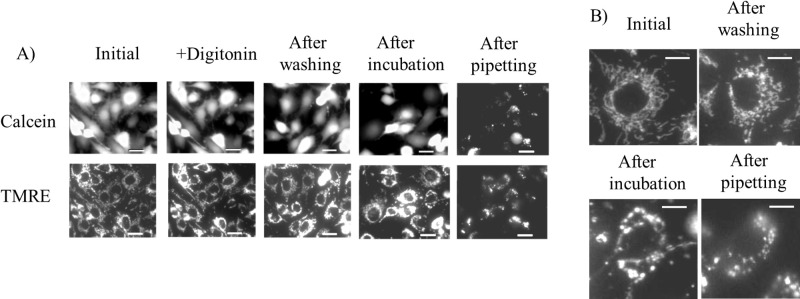
Fluorescence images of calcein and TMRE in C6 cells during iMIT. A) Calcein and TMRE fluorescence images of C6 cells cultured on GBD at each step of the iMIT procedure, shown in chronological order from Initial to After pipetting. Initial, before adding digitonin; +Digitonin, after adding digitonin; After washing, after washing digitonin; After incubation, after incubation for 10 min on ice; After pipetting, after pipetting the buffer that is bathing cells. B) Representative mitochondrial morphology in cells at each step of iMIT. Mitochondria were stained with TMRE. Scale bar=10 μm.

As shown in Figure 1A, calcein fluorescence was observed in all cells from before the addition of digitonin until after it was washed out, indicating that the plasma membrane remained intact while digitonin was present in the buffer. Although a few cells lost calcein fluorescence during the 10-minute incubation, TMRE retained mitochondria in all cells until the plasma membrane was disrupted by pipetting. These results suggest that neither the addition of digitonin nor the subsequent incubation caused mitochondrial damage. Furthermore, as shown in Figure 1B, intracellular mitochondria became fragmented and contracted during incubation in Tris isolation buffer. This morphological change may facilitate the release of mitochondria from the cell following plasma membrane disruption.

### Yields of mitochondria obtained using different isolation methods

**Figure 2:**
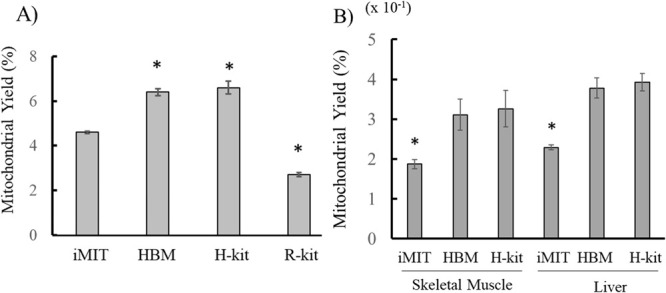
Comparison of mitochondrial yield among different isolation methods. A) Mitochondrial yields from cultured C6 cells. Yields are expressed as the ratio of mitochondrial protein to total cellular protein. Data are presented as mean±SEM (N=3). B) Mitochondrial yields from skeletal muscle and liver tissues. Yields are expressed as the ratio of mitochondrial protein to tissue wet weight. Data are presented as mean±SEM (N=3). *, p<0.05 vs. iMIT.

We compared four mitochondrial isolation methods—iMIT, HBM, H-kit, and R-kit—based on the mitochondrial yield from cultured cells. In this context, mitochondrial yield is defined as the ratio of the amount of mitochondrial protein obtained to the total amount of cellular protein used for isolation. As shown in Figure 2A, the mitochondrial yield from cultured C6 cells using the iMIT method was 4.6%. This yield was slightly lower than that obtained using the HBM and H-kit methods, and slightly higher than that obtained using the R-kit. However, overall differences among the methods were not substantial. A similar trend was observed when mitochondria were isolated from skeletal muscle and liver tissues using the iMIT, HBM, and H-kit methods, as shown in Figure 2B.

### Outer membrane integrity of isolated mitochondria

**Figure 3:**
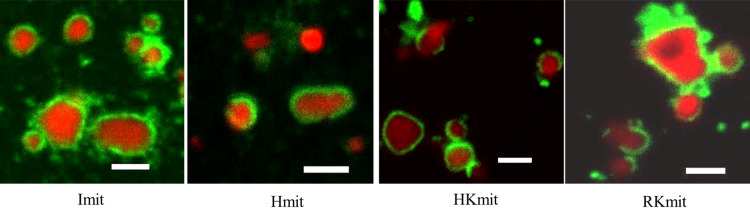
Membrane morphology of isolated mitochondria. Fluorescence images of the outer and inner membranes of individual mitochondria isolated from HeLa cells. Inner membranes were stained with MitoTracker Red (red) prior to isolation, and outer membranes were labeled with anti-TOM 20 antibodies (green) after isolation. Scale Bar=2 μm.

To assess the integrity of the outer mitochondrial membrane, we used STED microscopy to visualize both the outer and inner membranes of mitochondria isolated by different methods: iMIT (Imit), HBM (Hmit), H-kit (HKmit), and R-kit (RKmit). As shown in Figure 3, the inner membranes of most Imit were enclosed within intact outer membranes. In contrast, mitochondria isolated using HBM, HKmit, or RKmit often showed disrupted outer membranes, with inner membranes protruding beyond the outer membrane boundary. These observations indicate that the outer membrane remained largely intact in Imit, whereas it was frequently damaged in mitochondria isolated by the other methods.

**Figure 4:**
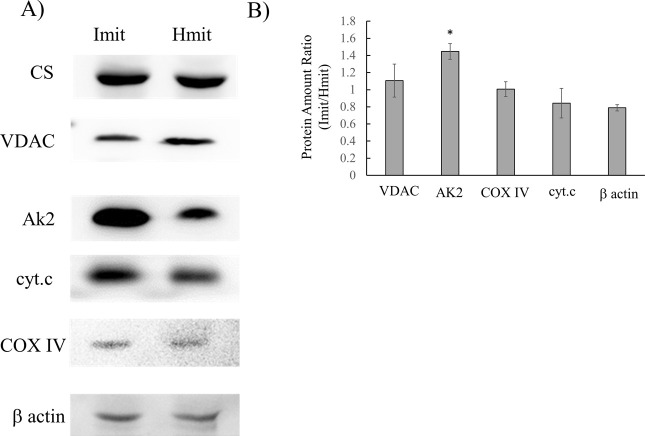
Western blotting analysis of mitochondrial proteins. Hmit and Imit were isolated from C6 cells. A) Western blot analysis of marker proteins from specific mitochondrial compartments: citrate synthase (CS, matrix), VDAC (outer membrane), AK2 (intermembrane space), cytochrome c (cristae space), COX IV (inner membrane), and β-actin (cytosol). B) Comparison of the relative levels of VDAC, AK2, cytochrome c, COX IV, and β-actin between Hmit and Imit. The vertical axis represents the ratio of the amount of each target protein in Imit to that in Hmit, defined as the R_target protein_ in the Materials and Methods section. The data were expressed as the mean±SEM (N=4 for VDAC, 6 for AK2, 5 for COX IV, 3 for cyt.c and β actin). *, p<0.05 vs. COX IV.

We further assessed outer membrane integrity by Western blotting. Because proteins located in the intermembrane space are expected to leak out when the outer membrane is disrupted during isolation, their abundance should be lower in damaged mitochondria than in intact ones. To test this, we quantified marker proteins localized in different mitochondrial compartments—intermembrane space, cristae space, outer membrane, inner membrane, and matrix—and compared Imit with Hmit. The proteins analyzed included adenylate kinase 2 (AK2, intermembrane space) [20], cytochrome c (cristae space) [21], citrate synthase (CS, matrix) [22], voltage-dependent anion channel (VDAC, outer membrane), cytochrome c oxidase subunit IV (COX IV, inner membrane), and β-actin (cytosol) (Figure 4A). As shown in Figure 4B, no significant differences were observed between Imit and Hmit in the levels of VDAC, cytochrome c, COX IV, or β-actin. However, the level of AK2 was lower in Hmit compared to Imit. As shown in Supplementary Figure S1, similar results were observed when comparing Imit with HKmit, with Imit showing higher levels of both cytochrome c and AK2. These results suggest that mitochondria isolated using iMIT retain better outer membrane integrity than those isolated using Hmit or HKmit, consistent with the findings from STED microscopy.

Although we have not yet quantitatively assessed the extent of contamination by other organelles in the mitochondrial fraction, Supplementary Figure S1B shows that the proportion CS relative to total protein does not differ significantly between mitochondria isolated using iMIT and those obtained with a commercially available kit. This suggests that the purity of mitochondria isolated by iMIT is comparable to that achieved with conventional isolation methods.

### Activity of isolated mitochondria

**Figure 5:**
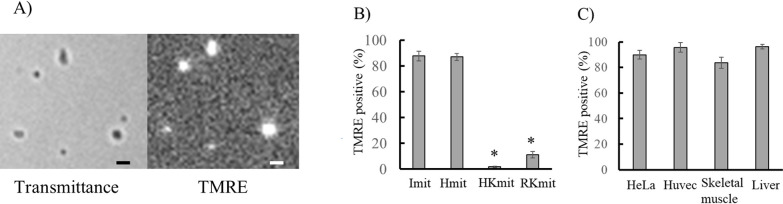
Percentages of polarized mitochondria isolated with iMIT. A) Microscopic images of isolated mitochondria adsorbed onto a coverslip. Left: transmitted light image; Right: TMRE fluorescence image of the same field. Mitochondria were isolated from HeLa cells. Scale bar=1 μm. B) Percentages of TMRE-positive mitochondria. Mitochondria were prepared from C6 cells using four different methods and bathed in Tris-isolation buffer containing 5 mM malate. Data were presented as mean±SEM (n=3 per group). *, p<0.05 vs Imit. C) Percentages of polarized mitochondria isolated from different sources using iMIT. Data were presented as mean±SEM (n=3). No significant differences were observed among the four groups.

Mitochondrial activity was evaluated by measuring the percentage of polarized mitochondria, defined as the proportion of particles exhibiting TMRE fluorescence (Figure 5A, right) among those approximately 1 μm in diameter observed in the transmitted light image (Figure 5A, left). The threshold for TMRE positivity was set at a fluorescence intensity ratio of 1.5 (mitochondrial TMRE fluorescence intensity/background fluorescence intensity), based on previous findings [23]. This threshold reflects the observation that 98% of depolarized mitochondria, treated with 5 μM carbonyl cyanide m-chlorophenyl hydrazone (CCCP), show a ratio below 1.5 (Supplementary Figure S2). The percentage of TMRE-positive mitochondria was 92±4% for Imit isolated from C6 cells, and 90±3%, 5±2%, and 10±2% for Hmit, HKmit, and RKmit, respectively (Figure 5B). These results indicate that Imit and Hmit retain significantly higher mitochondrial activity compared to HKmit and RKmit. High mitochondrial activity was also observed for Imit isolated from HeLa cells, HUVECs, skeletal muscle, and liver tissues (Figure 5C).

**Figure 6:**
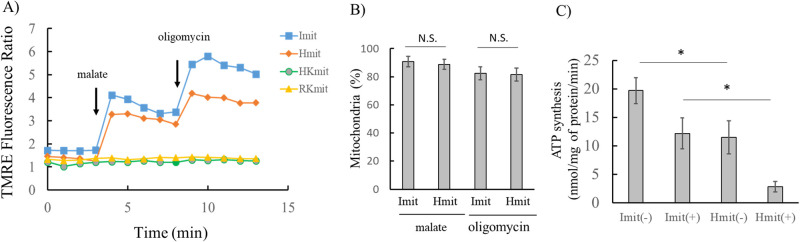
Electron transport chain activity in Imit, Hmit, HKmit, and RKmit. Mitochondria were isolated from C6 cells. A) Changes in TMRE fluorescence ratio following the addition of malate and oligomycin. The fluorescence ratio was averaged over 50 mitochondria for each measurement. Mitochondria were incubated in Tris-KCl buffer containing 0.5 mM ADP and 3 mM KH_2_PO_4_. Malate (1 mM) was added at t=3 min, and oligomycin (1 μM) was added at t=8 min. B) Percentages of mitochondria that exhibited polarization in response to malate addition and further polarization after oligomycin addition. C) ATP synthesis in Imit and Hmit, with and without oligomycin. (–) and (+) indicate absence and presence of oligomycin, respectively. Data are presented as mean±SEM (n=3). *, p<0.05.

Next, we analyzed individual mitochondria to evaluate changes in membrane potential. Both Imit and Hmit showed an increase in the TMRE fluorescence ratio following the addition of malate, with a further increase observed after the addition of oligomycin (Figure 6A). The noise level of the TMRE fluorescence ratio was less than 10%. Based on this threshold, a mitochondrion was considered significantly polarized by malate if the ratio of the mean TMRE fluorescence intensity during the 4 minutes after malate addition to the mean during the 4 minutes before exceeded 1.1. The same criterion was applied to assess the response to oligomycin.

The percentages of Imit that showed significant polarization were 91±4% upon the addition of malate and 82±5% upon subsequent addition of oligomycin (Figure 6B). These results indicate that the electron transport chain is active in most Imit and that FoF_1_-ATPase synthesizes ATP using the proton motive force generated by the chain. Similar responses were also observed in Hmit. In contrast, HKmit and RKmit did not exhibit any change in TMRE fluorescence (Figure 6A), suggesting a lack of membrane potential response.

As shown in Figure 6C, oligomycin significantly reduced ATP synthesis in both Imit and Hmit. Regardless of the presence or absence of oligomycin, ATP production was consistently higher in Imit than in Hmit. However, the extent of ATP reduction induced by oligomycin did not differ significantly between the two groups. These findings support the conclusion that both Imit and Hmit possess functional electron transport chains and FoF_1_-ATPase complexes capable of generating proton motive force and synthesizing ATP. The lower ATP production observed in Hmit may be attributed to its reduced level of adenylate kinase 2 (AK2), which facilitates efficient ATP generation.

### Effects of freeze-thaw on mitochondrial activity

**Figure 7:**
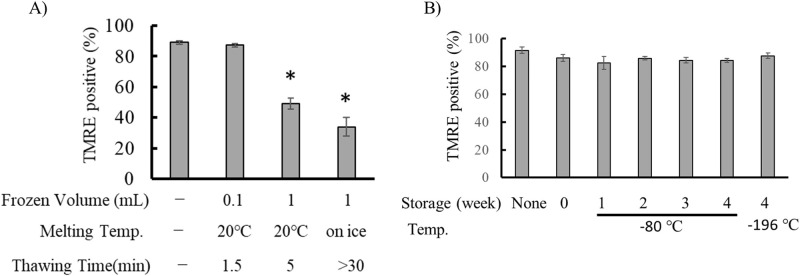
Effects of freeze-thaw treatment on the activity of isolated mitochondria. Mitochondria were isolated from C6 cells using the iMIT method. A) Effect of thawing rate on mitochondrial membrane potential. Mitochondrial suspensions (0.1 mL or 1.0 mL) were frozen in 3 mL tubes using liquid nitrogen and thawed either under running water at 20°C or on ice. The vertical axis indicates the percentage of TMRE-positive mitochondria. The leftmost bar represents unfrozen control mitochondria. *, p<0.05 vs unfrozen mitochondria. B) Effect of storage duration on mitochondrial activity. Imit samples from C6 cells were stored at –80°C or in liquid nitrogen. The vertical axis shows the percentage of TMRE-positive mitochondria. In each experiment, data were collected from more than 50 individual mitochondria. Results are presented as mean±SEM (n=3).

For long-term preservation, isolated mitochondria must be stored in a frozen state. However, freeze-thaw cycles can significantly impact mitochondrial activity [24,25]. In this study, we examined how the thawing rate of frozen mitochondria affects membrane potential. Mitochondrial suspensions were frozen in liquid nitrogen, and thawing rates were varied by altering the sample volume or thawing temperature. When 0.1 mL and 1.0 mL of mitochondrial suspension were frozen in 3.0 mL tubes, they thawed within 1.5 minutes and 5 minutes, respectively, under running water at 20°C. In contrast, thawing 1.0 mL of frozen mitochondria on ice required more than 30 minutes. As shown in Figure 7A, mitochondrial activity following freeze-thaw was strongly influenced by the thawing rate. When thawed within 1.5 minutes, the decrease in the percentage of polarized mitochondria was less than 10%. Furthermore, even after storage at –80°C or –196°C for one month, mitochondrial activity did not significantly decline compared to that observed when thawed immediately after freezing (Figure 7B).

## Discussion

**Figure 8:**
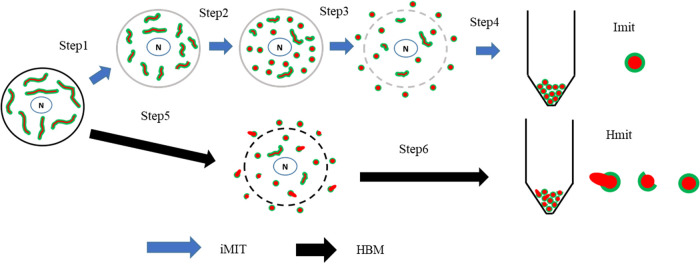
Schematic illustration of iMIT and HBM for mitochondrial isolation. Solid black lines denote firm plasma membranes, while solid gray lines indicate weak ones. Broken gray lines indicate disrupted plasma membranes. Green lines represent the outer mitochondrial membranes. The red indicates the inner mitochondrial membrane. The blue line denotes the nuclear envelope, and the N indicates the nucleus. In the initial state, plasma membranes were firm and mitochondria were elongated and surrounded by outer membranes. Steps 1-4 are for iMIT. Step 1: Incorporation of digitonin into the plasma membrane, followed by removal of free digitonin from the buffer. After Step 1, plasma membranes become weak. Step 2: Incubation of cells in the buffer for isolated mitochondria. After Step 2, mitochondria shrink and fragment in cells. Step 3: Selective disruption of plasma membranes with pipetting. After Step 3, the intact, spherical mitochondria are released from the cells. Step 4: Collection of mitochondrial fractions with differential centrifugation. Steps 5 and 6 are for HBM. Step 5: Homogenization of cells. After Step 5, the outer membranes of the mitochondria are significantly damaged. Step 6: Same as Step 4.

We have developed a novel method (iMIT) to isolate intact mitochondria without damaging them (Figure 8). iMIT consists of four steps: Step 1) selectively weakening the strength of the plasma membrane, Step 2) shortening the mitochondria in the cell, Step 3) disrupting the plasma membrane by gentle pipetting, and Step 4) collecting the mitochondria with differential centrifugation.

In a previous study, we used streptolysin O to weaken the plasma membrane [26]. However, this approach did not sufficiently reduce membrane integrity, resulting in a low mitochondrial yield and limiting the number of feasible experiments. Attempts to increase the yield by vigorous pipetting led to the isolation of damaged mitochondria, and intact mitochondria were not predominant in the final preparation. To address this, we used digitonin in the present study to selectively weaken the plasma membrane. During the 10-minute incubation following digitonin removal, mitochondria changed from a networked morphology to a short, spherical shape. This transformation may facilitate mitochondrial isolation, as shorter mitochondria are more likely to escape from the cell when the plasma membrane is compromised. Accordingly, we applied gentle pipetting to selectively disrupt the plasma membrane of cells containing shortened mitochondria. As a result, we successfully isolated a mitochondrial population comparable in quantity to that obtained by conventional methods, with most mitochondria retaining both outer and inner membranes intact.

Digitonin has been reported to disrupt the plasma membrane at high concentrations [15] and to increase its permeability to small molecules at low concentrations [27,28], although mitochondria typically do not exit the cell under these conditions. In contrast, our method did not increase plasma membrane permeability to small molecules, at least while digitonin remained present outside the cell. This may be due to our use of a low digitonin concentration, applied at low temperature for a short duration, with minimal mechanical stimulation of the cells. While the plasma membranes of all cells remained intact before digitonin was removed, increased permeability was observed in a small number of cells immediately after digitonin removal, and in a similar proportion of cells following a 10-minute incubation. It is important to maintain low plasma membrane permeability while digitonin is still present outside the cell. Previous studies have shown that adding digitonin to isolated mitochondria has no effect at concentrations up to 0.001%, but damages the outer membrane at concentrations of 0.003% or higher [17]. Although the 30 μM digitonin used in this study corresponds to 0.004%, it was washed out before any increase in plasma membrane permeability occurred. Therefore, we consider the amount of digitonin that reached the mitochondria to be negligible and unlikely to affect mitochondrial activity.

In conventional mitochondrial isolation methods, mitochondria are extracted either by disrupting the plasma membrane through homogenization or other mechanical forces, or by solubilizing the membrane using detergents. In the former approach, mitochondria that exit the cell early are repeatedly exposed to mechanical stress in the extracellular environment, often resulting in severe damage, particularly to the outer membrane. In the latter case, detergents that enter the cell can directly affect the mitochondrial outer membrane, likewise causing damage. In the present study, damage to the outer membrane was frequently observed in mitochondria isolated by homogenization or by disrupting the plasma membrane in the presence of chemical reagents. To avoid repeated mechanical stress on mitochondria that have exited the cell, an alternative method using the N_2_ cavitation technique has been employed [16,29]. This approach has been reported to isolate highly active mitochondria even from cultured neurons and other cell types [16]. However, this method does not selectively weaken the plasma membrane, and potential damage to the mitochondrial outer membrane still requires careful evaluation. Additionally, the rapid isolation of mitochondria is essential for preserving mitochondrial activity [30]. The iMIT method fulfills this requirement, taking less than 45 minutes in total, with under 30 minutes from cell disruption by pipetting to mitochondrial collection.

The cryopreservation of isolated mitochondria has been investigated with a focus on preservation solutions. Nukala et al. [25] added DMSO as a cryoprotective agent and obtained mitochondria that retained approximately 60% of their state III respiration rate compared to pre-freezing levels, despite significant damage to the outer membrane. Yamaguchi et al. [31] used trehalose during freeze-thaw cycles to reduce outer membrane damage; however, they also observed a decrease in mitochondrial bioenergetic function. In our study, we successfully minimized the loss of bioenergetic activity by rapidly thawing cryopreserved mitochondria in a sucrose-containing buffer.

## Conclusion

iMIT is a method for isolating mitochondria with minimal damage to both the outer and inner membranes. This is achieved by selectively weakening the plasma membrane and gently disrupting it, while allowing mitochondria to undergo spontaneous contraction and fragmentation within the cell. iMIT has the potential to make a significant contribution to both mitochondrial research and mitochondria-based therapeutic applications.

## Conflict of interest

YOh was a co-inventor named on patent applications by LUCA Science Inc. The terms of this arrangement have been reviewed and approved by the Tokyo University of Agriculture and Technology, Japan, in accordance with its conflict-of-interest policies.

## Ethics statement

Mitochondria isolation from animals was conducted with the approval of the University of Agriculture and Technology (Approval No. 31–54).

## Author contributions

AO, JN, AOg, and TM conducted the experiments and analyzed the results. IO and YO designed the experiments and prepared the manuscript. All authors approved the final manuscript.

## Data availability

The evidence data generated and/or analyzed during the current study are available from the corresponding author on reasonable request.

## Abbreviation

AK2: Adenylate kinase 2
COX IV: subunit IV of cytochrome c oxidase
CS: citrate synthase
cyt.c: cytochrome c
GBD: glass-base dish
H-kit: Mitochondria isolation procedure using the homogenization based-method with a mitochondrial isolation kit
HKmit: mitochondria isolated with H-kit
HBM: the procedure for mitochondria isolation with the homogenization-based method
Hmit: mitochondria isolated with HBM
iMIT: the novel method for mitochondrial isolation reported here
Imit: mitochondria isolated with iMIT
R-kit: Mitochondria isolation procedure using a reagent-based method with a mitochondrial isolation kit
RKmit: mitochondria isolated with R-kit, Huvec, human umbilical vein endothelial cells
TMRE: tetramethyl rhodamine ethyl ester
VDAC: voltage-dependent anion channel.
