# Supplementary material for: A method for isolating and cryopreserving intact mitochondria with improved integrity and functionality
Source: Biophys Physicobiol. 2025 May 16;22(2):e220012. doi: 10.2142/biophysico.bppb-v22.0012 (PMC12226260; doi:10.2142/biophysico.bppb-v22.0012)
Supplement: Supplementary file 1 — Supplementary Materials [file 22_e220012_1.pdf]

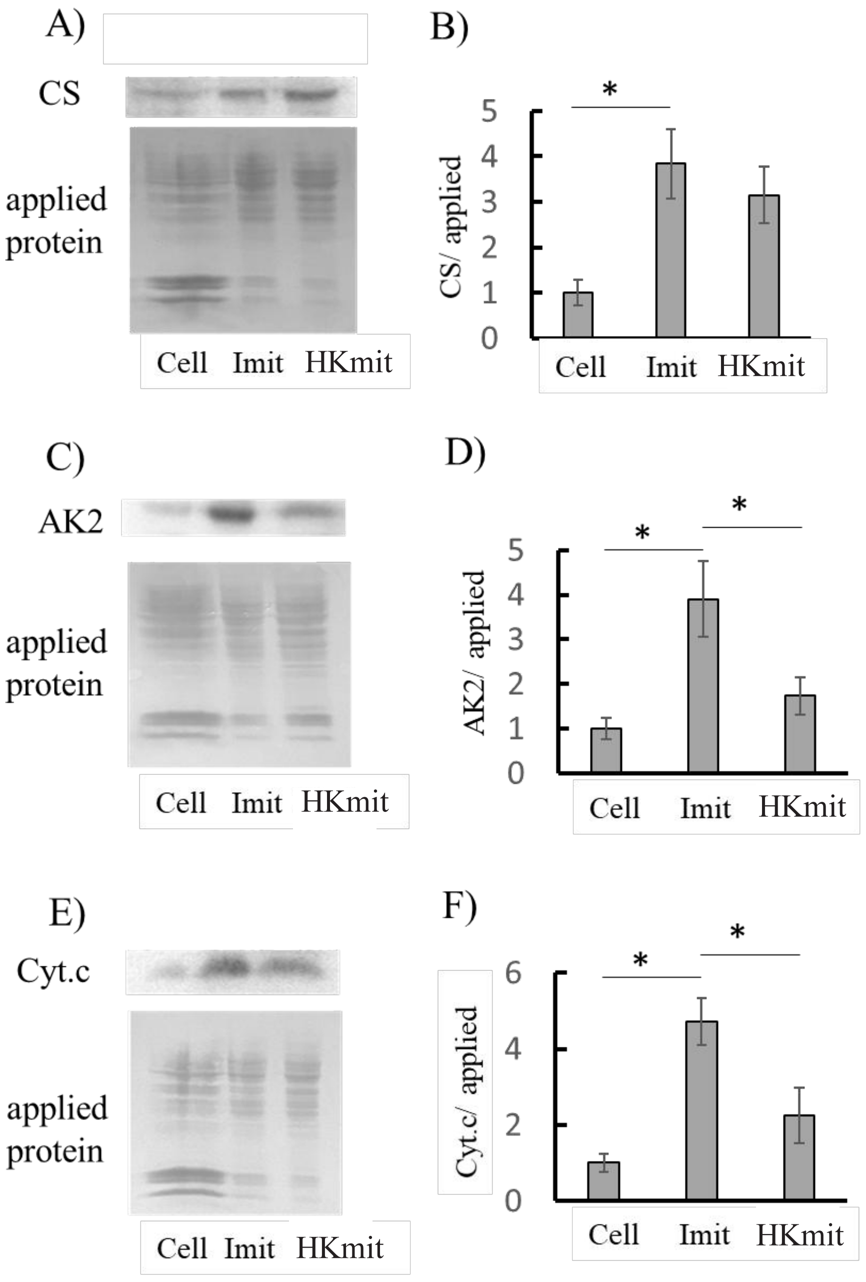

Supplementary Figure S1. Western blot analysis of CS, AK2, and cytochrome c.
Western blotting and Ponceau S staining were performed to assess protein levels. **A, B)** Citrate synthase (CS); **C, D)** adenylate kinase 2 (AK2); **E, F)** cytochrome c (cyt. c). **B, D, F)** Relative protein levels. Band intensities from Western blots were normalized to the total intensity of Ponceau S stained bands. The normalized value for C6 cells was set to 1.0. Data are presented as mean ± SEM (N = 3 for CS and AK2; N = 4 for cyt. c). *$p < 0.05$.

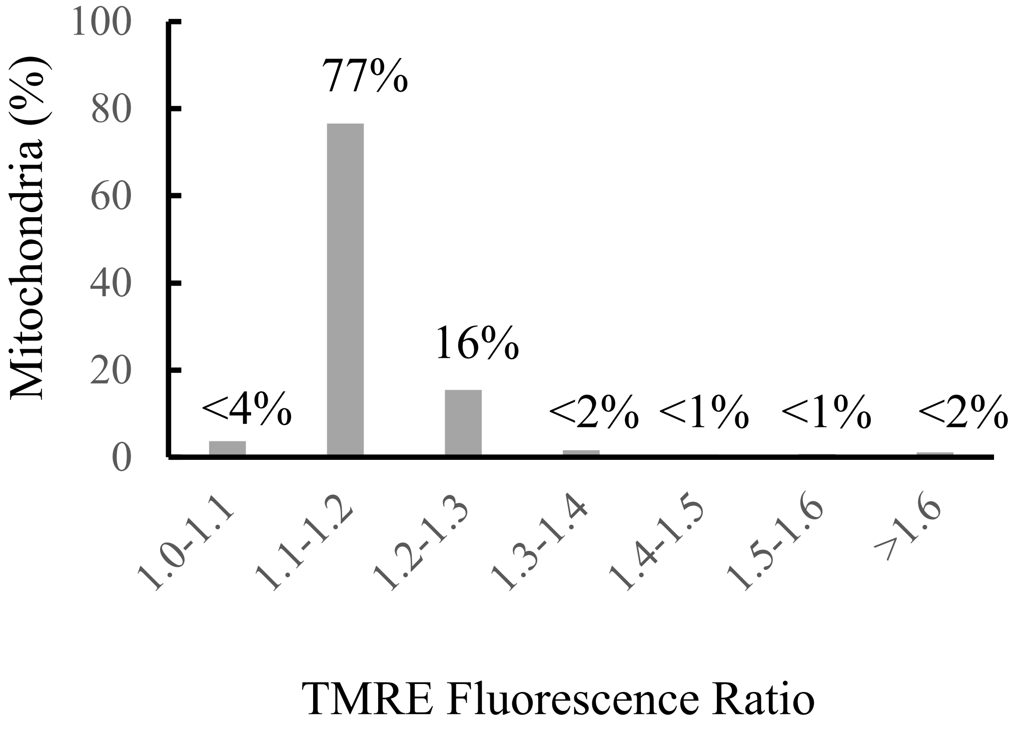

Supplementary Figure S2. Distribution of TMRE fluorescence ratios in depolarized mitochondria.
Imit isolated from C6 cells were depolarized using 5 µM carbonyl cyanide m-chlorophenyl hydrazine (CCCP). TMRE fluorescence intensity for each mitochondrion was normalized to the background TMRE fluorescence intensity. A total of 509 mitochondria were analyzed to determine the distribution of fluorescence ratios. Among them, 98% exhibited a TMRE fluorescence ratio below 1.5.
